# Cross‐sectional analyses of factors associated with the presence and aggravation of chronic insomnia by symptom subtypes

**DOI:** 10.1002/pcn5.184

**Published:** 2024-03-25

**Authors:** Masumi Osao, Isa Okajima, Yuichi Inoue

**Affiliations:** ^1^ Yoyogi Sleep Disorder Center Tokyo Japan; ^2^ Department of Psychological Counseling, Faculty of Humanities Tokyo Kasei University Tokyo Japan; ^3^ Japan Somnology Center, Neuropsychiatric Research Institute Tokyo Japan; ^4^ Department of Somnology Tokyo Medical University Tokyo Japan

**Keywords:** chronotype, insomnia, pre‐sleep arousal, sleep reactivity, sleep‐related attitudes and beliefs

## Abstract

**Aim:**

The aim of this study was to investigate the association of psychological and chronobiological factors with the presence and severity of chronic insomnia by symptom subtypes and their impacts on daytime dysfunctions.

**Methods:**

Participants of the present web‐based epidemiological study were classified as follows: difficulty initiating sleep (DIS) (*n* = 91); difficulty maintaining sleep (DMS) (*n* = 13); early morning awakening (EMA) (*n* = 48); DIS + DMS (*n* = 67); DIS + EMA (*n* = 23); DMS + EMA (*n* = 24); triplet of DIS, DMS, and EMA symptoms (TRP) (*n* = 69); and normal sleepers (*n* = 4590). The Insomnia Severity Index (ISI), Hospital Anxiety and Depression Scale (HADS), Munich Chronotype Questionnaire (MCTQ), insomnia‐related psychological measures (including the Ford Insomnia Response to Stress Test [FIRST] and the Dysfunctional Beliefs and Attitudes about Sleep Scale [DBAS]), and the cognitive and somatic domains of the Pre‐Sleep Arousal Scale (PSAS) were evaluated.

**Results:**

The presence of DIS and DIS + DMS were significantly associated with an evening preference, and EMA and EMA + DMS with a morning preference, while TRP showed no significant association with either chronotype. The increase in DBAS scores was associated with higher ISI scores in all subtypes. Meanwhile, the associations of each psychological measure varied among insomnia subtypes, with the association of PSAS cognitive arousal to DIS and PSAS somatic arousal to both DMS + EMA and TRP. Pathological HADS score was associated with all subtypes.

**Conclusion:**

Chronotypes may be associated with the presence of some insomnia subtypes; however, only psychological factors were speculated to contribute to the aggravation of all subtypes. All insomnia subtypes possibly contribute to the formation of depression.

## Graphical abstract

Subtypes including difficulty initiating sleep (DIS) tended to be evening preference, while those including early morning awakening (EMA) tended to be morning preference. There was no chronotypic tendency in the group including all subtypes or the group including DIS and EMA. The psychological factors associated with each subtype differed; DIS was significantly associated with PSAS cognitive arousal and TRP with PSAS somatic arousal, indicating that the psychological factors associated with each subtype differed.

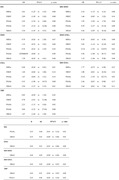

## INTRODUCTION

Chronic insomnia disorder is diagnosed when an individual exhibits nocturnal phenotypic symptoms, including difficulty initiating sleep (DIS), difficulty maintaining sleep (DMS), and early morning awakening (waking up earlier than the desired schedule in the morning) (EMA), at least three nights per week, with resultant daytime symptoms, and persists for 3 months or longer.^1^ Reportedly, 5.8%–17.5% of the general population has insomnia disorder and 26.6% have insomnia symptoms.^2^, ^3^ The pathological mechanism of chronic insomnia has been studied from both physiological and psychological aspects. For instance, insomnia is widely accepted to be a major risk for the development of depression and anxiety.^4^, ^5^, ^6^

In a previous study, chronotype was identified as a factor that negatively affected insomnia. Lack et al.^7^ observed significant improvement in subjective DIS symptoms in people with insomnia following experimental bright‐light exposure early in the morning. Furthermore, exposure of patients with EMA to bright light in the evening showed significant delays in the rectal temperature rhythm and melatonin secretion profile, with improvements in subjective and objective sleep measures.^8^ Thus, DIS and EMA may be associated with a circadian‐phase delay and sleep‐phase advance, respectively. On the other hand, Bjorøy et al.^9^ reported that the group with a triplet of DIS, DMS, and EMA symptoms (TRP) exhibited a morningness chronotype.^9^ However, similar studies on this aspect have not been conducted since then, and the relationships between chronotypes of other insomnia symptom combinations have not been elucidated. Meanwhile, sleep reactivity, dysfunctional beliefs, and hyperarousal have also been proposed as factors affecting insomnia.^10^, ^11^, ^12^ However, it remains unclear in which process of the disorder these psychological factors play pathological roles among respective insomnia subtypes.

To clarify the above issues, a comprehensive investigation on the involvement of chronotype and sleep‐related psychological measures for the development and aggravation of each insomnia subgroup, including mixed types by using already authorized measures, would be necessary. To estimate an individual's chronotype or a morningness–eveningness preference reflecting one's circadian rhythm characteristics, the Munich Chronotype Questionnaire (MCTQ)^13^, ^14^ has generally been used. The validity of the Ford Insomnia Response to Stress Test (FIRST)^10^ has already been established in measuring the effect of psychological stress on the development of insomnia. The Dysfunctional Beliefs and Attitudes about Sleep Scale (DBAS) has been accepted as useful for measuring sleep‐related false cognitions (e.g., faulty beliefs and appraisals, unrealistic expectations, perceptual and attention bias).^15^ Furthermore, a decrease in somatic and cognitive hyperarousal evaluated with the Pre‐Sleep Arousal Scale (PSAS)^11^ has been reportedly associated with the improvement of insomnia symptoms following cognitive behavioral therapy for insomnia (CBT‐I).^16^, ^17^ Puzino et al. also reported that PSAS correlated with insomnia severity.

Considering this information, we designed this cross‐sectional study to epidemiologically investigate the role of chronotype and sleep‐related psychological measures in the presence and severity of respective insomnia subtypes using the abovementioned measures, controlling for participant age and sex. Subsequently, we estimated the relationship between each subtype and the development of depression and anxiety in individuals with insomnia.

## METHODS

The study protocol was approved by the Ethics Committee of Tokyo Medical University, Tokyo, Japan (registration no. 3307). All study participants provided informed consent prior to participation, in accordance with the Declaration of Helsinki.

### Participants

The study was conducted as a part of a web‐based, cross‐sectional questionnaire survey on sleep health in the Japanese general population in February 2015. Participants were recruited by Rakuten Research Inc. (Tokyo, Japan), an online marketing research company holding panels with approximately 2.3 million Japanese people. An e‐mail containing a link to the online questionnaire was randomly circulated to individuals throughout Japan who were stratified by district, sex, and age. The age of the participants ranged from 20 to 69 years. A total of 10,000 responses were received. We excluded participants who did not provide complete answers (*n* = 1035) and those who were currently receiving treatment for a disease (*n* = 4040). Overall, 4925 participants (2364 men, 2561 women; 44.53 ± 14.39 years) were included in the analyses.

### Measures

First, demographic data (age and sex) were collected in this questionnaire. Subsequently, the respondents were asked to answer questions related to the presence or absence of subjective insomnia and its constituent symptoms, including DIS, DMS, and EMA, based on the diagnostic criteria for chronic insomnia disorder as outlined in the International Classification of Sleep Disorders‐3 (ICSD‐3).^1^ Participants were also asked whether sleep disturbances and their associated daytime symptoms persisted at least three times per week and had been present for at least 3 months.^1^

Insomnia severity as well as the psychological and chronotypic status were self‐reported with the following measures.

#### Insomnia Severity Index

The Insomnia Severity Index (ISI) is a validated, seven‐item, self‐reported questionnaire that assesses the severity of insomnia. A summed score is calculated ranging from 0 to 28, with higher scores indicating severe insomnia symptoms.^18^, ^19^ The pathological cutoff point for the ISI is set at 10 points.^19^

#### FIRST

The FIRST is a validated, nine‐item, self‐reported questionnaire that assesses sleep reactivity to stress (i.e., hyperarousal caused by a stressful event). A summed score is calculated (range, 9–36), with higher scores indicating a larger sleep reactivity.^10^, ^20^ Since the FIRST does not have a cutoff point, we dichotomized samples by the mean score.

#### DBAS

The DBAS‐16 scale is a validated, 16‐item, self‐reported questionnaire. A higher summed score (range, 0–160) indicates more dysfunctional beliefs about sleep.^19^, ^21^ Because a certain cutoff score is not set for DBAS, we dichotomized samples by the mean score.

#### PSAS

The PSAS is a validated, 16‐item, self‐reported questionnaire that assesses pre‐sleep arousal.^22^, ^23^ This scale is constructed with two eight‐item domains: somatic arousal (i.e., physiological) (PSASs) and cognitive arousal (PSASc). A higher summed score of each subscale (range: 8–40) indicates higher pre‐sleep arousal. PSAS also does not have a cutoff score, and so we dichotomized samples by the mean score.

#### Munich Chronotype Questionnaire

The Munich Chronotype Questionnaire (MCTQ) was used to determine the participants' chronotype or a morningness–eveningness preference reflecting one's circadian rhythm characteristics.^13^, ^14^ The chronotype was assessed using a sleep debt‐corrected Midpoint of Sleep on Free Days (MSFsc) score.^14^ Since MSFsc does not have a cutoff point, we dichotomized samples by the mean score.

#### Hospital Anxiety and Depression Scale

The Hospital Anxiety and Depression Scale (HADS) was used to evaluate the states of depression and anxiety.^24^, ^25^ The scale is constructed with Anxiety (HADSa) and Depression (HADSd) subscales. The higher the summed scores of each subscale (range: 1–28), the higher the levels of anxiety and depression. The cutoff score for both HADSa and HADSd for pathological anxiety and depression, respectively, is set at 8 points.^25^

**Figure 1:**
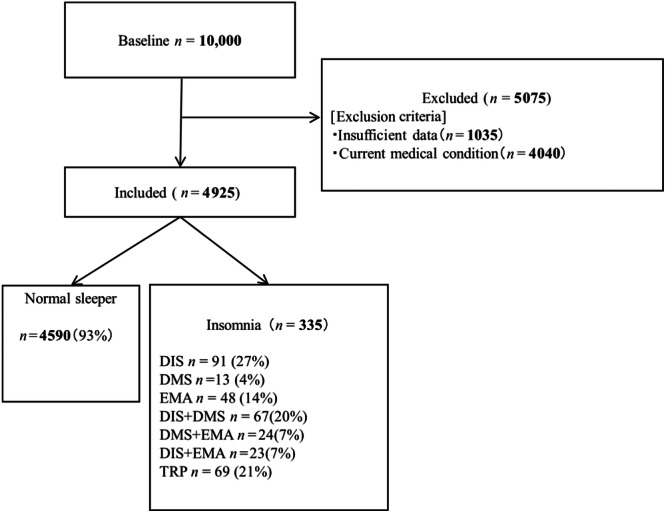
Participant enrollment flowchart for this study. DIS, difficulty initiating sleep; DMS, difficulty maintaining sleep; EMA, early morning awakening; TRP; triplet of DIS, DMS, and EMA symptoms.

In this study, people with insomnia were classified by the pattern of all phenotype combinations in accordance with previous studies.^9^, ^26^ The following eight subtypes proceeded to the subsequent analyses in this study: DIS (participants with only DIS), DMS (participants with only DMS), EMA (participants with only EMA), DIS + DMS (participants with both DIS and DMS), DMS + EMA (participants with both EMA and DMS), DIS + EMA (participants with both DIS and EMA), TRP (participants with a triplet of DIS, DMS, and EMA symptoms), and normal sleepers (NS; i.e., those with ISI < 10) (Figure 1).

### Statistical analysis

To examine the differences in sociodemographic, chronotypic, and psychological backgrounds in addition to disease severity among respective insomnia subtypes, we performed an analysis of variance (ANOVA), using subtypes as the independent variable and age and MSFsc, FIRST, PSASs, PSASc, and DBAS scores as the dependent variables. A *χ* ^2^ test followed by a residual analysis was performed for sex comparison. When main and/or interaction effects were observed in the ANOVA, Bonferroni corrections for estimating *p* values were performed as post hoc analyses. To examine the magnitude of differences between groups, Hedges's *g* was calculated as a measure of effect size.^27^ Logistic regression analyses were performed to examine the factors associated with the presence of respective insomnia subtypes, using MSFsc, FIRST, PSASs, PSASc, and DBAS scores as independent variables and the presence of each subtype as the dependent variable. Similar to previous studies,^28^, ^29^ age and sex were used as adjustment variables. Moreover, to examine the factors associated with the severity of insomnia manifested on ISI in each subtype, a stepwise method of multiple regression analysis was performed using descriptive measures (FIRST, PSASs, PSASc, DBAS, and MSFsc scores) as independent variables, ISI scores as a dependent variable, and the demographic variables as adjustment variables. Finally, to examine the subtypes that influence anxiety and depression, logistic regression analyses were performed using the presence of pathological anxiety or depression as dependent variables and respective subtypes as independent variables.

The data were analyzed using SPSS Version 23.0 (IBM). A *p*‐value < 0.05 was considered statistically significant.

## RESULTS

### Characteristics of chronotypes and cognitive‐behavioral factors among respective insomnia symptom subtypes

Of the 4925 included participants, 335 (7%) were determined to have chronic insomnia. Of these, 91 (27%) had DIS (mean age 39.98 ± 13.76 years, 36 males, 55 females), 13 (4%) had DMS (45.77 ± 17.10 years, 4 males, 9 females), 48 (14%) had EMA (44.63 ± 12.35 years, 31 males, 17 females), 67 (20%) had DIS + DMS (41.57 ± 14.90 years, 26 males, 41 females), 24 (7%) had DMS + EMA (47.58 ± 11.61 years, 14 males, 10 females), 23 (7%) had DIS + EMA (37.17 ± 14.23 years, 11 males, 12 females), and 69 (21%) had TRP (44.33 ± 12.82 years, 28 males, 41 females) (Table 1).

**Table 1:** Clinical descriptive variables.

	Total		NS		DIS		DMS		EMA		DIS + DMS		DMS + EMA		DIS + EMA		TRP		*F* value or *χ* ^2^ value	*p* Value	Post hoc multiple comparison
	*n* = 4925		*n* = 4590		*n* = 91		*n* = 13		*n* = 48		*n* = 67		*n* = 24		*n* = 23		*n* = 69				
					27%		4%		14%		20%		7%		7%		21%				
Rate^a^	Mean	SD	Mean	SD	Mean	SD	Mean	SD	Mean	SD	Mean	SD	Mean	SD	Mean	SD	Mean	SD			
Age (years)	44.53	(14.39)	44.68	(14.42)	39.98	(13.76)	45.77	(17.10)	44.63	(12.35)	41.57	(14.90)	47.58	(11.61)	37.17	(14.23)	44.33	(12.82)	3.28	0.00	No significant difference
Sex (men/%)	2364	48%	2214	48%	36	40%	4	31%	31	65%	26	39%	14	58%	11	48%	28	41%	12.80	0.05	In EMA, men < women
ISI (points)	4.20	(3.59)	3.53	(2.55)	12.35	(2.54)	12.15	(2.19)	12.54	(2.80)	13.27	(3.22)	14.88	(3.80)	13.22	(2.73)	15.06	(3.79)	738.67	0.00	DIS, DMS, EMA, DIS + DMS, DMS + EMA, DIS + EMA, TRP > NS TRP > DIS, DMS, EMA, DIS + DMS DMS + EMA > DIS, EMA
MSFsc	3.70	(1.55)	3.65	(1.49)	4.78	(1.89)	3.41	(0.98)	3.83	(1.96)	5.07	(2.14)	2.68	(1.25)	5.15	(2.45)	4.30	(2.17)	24.45	0.00	DIS, DIS + DMS, DIS + EMA, DMS + EMA, TRP > NS DIS + DMS > EMA, DMS + EMA DIS + DMS > DMS DIS > EMA, DIS + EMA DIS + EMA > DMS + EMA
FIRST(points)	17.55	(6.21)	17.06	(5.91)	25.23	(6.38)	22.00	(5.46)	22.17	(7.26)	23.69	(6.72)	23.50	(6.31)	23.35	(5.68)	25.84	(5.49)	77.38	0.00	DIS, EMA, DIS + DMS, DMS + EMA, DIS + EMA, TRP > NS EMA > TRP
PSASs(points)	11.86	(4.36)	11.45	(3.87)	17.59	(6.05)	12.15	(2.34)	15.46	(5.91)	17.58	(6.25)	19.17	(6.16)	17.52	(4.85)	19.03	(7.20)	122.00	0.00	DIS, EMA, DIS + DMS, DMS + EMA, DIS + EMA, TRP > NS DIS > DMS DIS + DMS. DIS + EMA, DMS + EMA > DMS
PSASc (points)	13.54	(6.11)	12.76	(5.17)	25.19	(7.66)	20.77	(5.49)	20.00	(7.58)	24.18	(7.66)	23.88	(7.24)	24.74	(7.75)	26.38	(8.26)	240.07	0.00	DIS, DMS, EMA, DIS + DMS, DMS + EMA, DIS + EMA, TRP > NS DIS, DIS + DMS, DIS + EMA, DMS + EMA > DMS DMS + EMA > EMA
DBAS (points)	49.37	(31.50)	47.38	(30.50)	78.22	(30.75)	61.92	(26.70)	72.48	(32.23)	79.33	(31.58)	74.13	(35.27)	76.83	(32.80)	78.35	(35.20)	47.86	0.00	DIS, DIS + DMS, DIS + EMA, DMS + EMA, TRP > NS
HADSa (points)	4.70	(3.59)	4.39	(3.30)	9.40	(4.34)	6.54	(4.03)	8.29	(4.40)	9.45	(4.05)	8.96	(4.09)	10.43	(5.02)	10.68	(4.70)	119.71	0.00	DIS, EMA, DIS + DMS, DMS + EMA, DIS + EMA, TRP > NS DISLEMA > DMS TRP > DMS, EMA
HADSd (points)	7.46	(3.58)	7.28	(3.49)	10.44	(3.97)	8.92	(2.50)	9.85	(3.33)	10.22	(3.83)	9.17	(3.56)	11.39	(3.86)	10.29	(3.61)	36.74	0.00	DIS, EMA, DIS + DMS, DMS + EMA, DIS + EMA, TRP > NS

*Note*: Values are expressed as mean ± SD for continuous variables and as number and percentage (%) for categorical variables.

Abbreviations: DBAS, Dysfunctional Beliefs and Attitudes about Sleep Scale; DIS, difficulty initiating sleep; DMS, difficulty maintaining sleep; EMA, early morning awakening; FIRST, Ford Insomnia Response to Stress Test; HADSa, Hospital Anxiety and Depression Scale–Anxiety subscale; HADSd, Hospital Anxiety and Depression Scale–Depression subscale; ISI, Insomnia Severity Index; MSFsc, sleep debt‐corrected Midpoint of Sleep on Free Days; NS, normal sleeper; PSASs, Pre‐sleep Arousal Scale–Somatic; PSASc, Pre‐sleep Arousal Scale–Cognitive; SD, standard deviation; TRP, triplet of DIS, DMS, and EMA symptoms.

^a^ Rate: The percentage of each subgroup among all insomniacs.

Statistical test results for the comparisons of descriptive variables are shown in Table 1. Participants with DIS were significantly younger than NS participants, which was confirmed by post hoc multiple comparisons. The *χ* ^2^ test for sex distribution revealed a significant difference among all groups. Residual analysis showed that the EMA group had a significant male predominance. Significant differences were observed in the ISI, MSFsc, FIRST, PSASs, PSASc, DBAS, HADSa, and HADSd scores among the groups.

Post hoc multiple comparison tests among each subtype group showed that the ISI scores were significantly higher in all insomnia groups than in NS participants. The TRP group scored significantly higher than the DIS, DMS, EMA, and DIS + DMS groups. The DMS + EMA group scored significantly higher than the DIS and EMA groups.

The MSFsc scores in the DIS, DIS + DMS, DIS + EMA, DMS + EMA, and TRP groups were significantly higher than that in the NS group. In addition, the DIS + DMS group had significantly higher scores than the EMA and DMS + EMA groups. Conversely, the DMS + EMA group scored significantly lower than the NS group, while the DIS + DMS group scored significantly higher than the DMS group. Conversely, the EMA and DMS + EMA groups had significantly lower scores than the DIS group. The DMS + EMA group also had significantly lower scores than the DIS + EMA and TRP groups. The EMA group scored significantly lower than the DIS + EMA group. The DMS group had significantly lower scores than the DIS + EMA and DIS + DMS groups. Finally, the DIS + EMA group scored higher than the DMS + EMA group. However, no significant differences were found among the other groups.

The FIRST scores of all insomnia symptom subtypes were significantly higher than the NS group, except for the DMS group. Among the subtypes, the score of the EMA group was significantly higher than that of the TRP group.

As for PSASs, all insomnia symptom subtypes scored significantly higher than the NS group, except for the DMS group. In addition, the DIS group scored significantly higher than the DMS group. Furthermore, the DIS + DMS, DIS + EMA, and DMS + EMA groups scored significantly higher than the DMS group.

Regarding PSASc, scores for all subtypes were significantly higher than those for the NS group. In addition, the DIS, DIS + DMS, DIS + EMA, and DMS + EMA groups scored significantly higher than the DMS group. Furthermore, the DMS + EMA group scored significantly higher than the EMA group.

The DBAS scores of all insomnia symptom subtypes were significantly different from the NS group, except for the DMS group.

The HADSa scores of all insomnia symptom subtypes were significantly higher than the NS group, except for the DMS group. In addition, the DIS + EMA group scored higher than the DMS group, and the TRP group scored higher than the DMS and EMA groups.

Finally, the HADSd scores of all insomnia symptom subtypes were significantly higher than the NS and DMS + EMA groups, except for the DMS group.

### Factors associated with the presence of respective insomnia subtypes

The results of logistic regression analyses of factors associated with the presence of respective insomnia subtypes after adjusting for demographic variables are presented in Table 2. The scores of MSFsc (odds ratio [OR]: 2.01, 95% confidence interval [CI]: 1.25–3.23, *p* < 0.001), FIRST (OR: 2.89, 95% CI: 1.49–5.62, *p* < 0.001), PSASs (OR: 2.43, 95% CI: 1.34–4.40, *p* < 0.001), PSASc (OR: 7.23, 95% CI: 3.16–16.56, *p* < 0.001), and DBAS (OR: 2.54, 95% CI: 1.43–4.51, *p* < 0.001) were significantly associated with the presence of DIS subtype, with the MSFsc in this group showing a significant association with the positive direction (i.e., eveningness preference). On the other hand, the MSFsc (OR: 0.44, 95% CI: 0.24–0.81, *p* = 0.01), PSASc (OR: 4.53, 95% CI: 1.98–10.37, *p* < 0.001), and DBAS (OR: 2.56, 95% CI: 1.27–5.15, *p* = 0.01) scores were significantly associated with the presence of EMA subtype, with the MSFsc showing a negative direction (i.e., morningness preference). The scores of MSFsc (OR: 2.34, 95% CI: 1.33–4.12, *p* < 0.001), PSASs (OR: 1.95, 95% CI: 1.02–3.74, *p* = 0.04), PSASc (OR: 8.70, 95% CI: 3.27–23.14, *p* < 0.001), and DBAS (OR: 4.56, 95% CI: 2.05–10.16, *p* < 0.001) were also significantly associated with the DIS + DMS subtype. Meanwhile, not the MSFsc but the PSASs score was significantly associated with DIS + EMA (OR: 15.43, 95% CI: 1.95, 122.37, *p* = 0.01). Regarding DMS + EMA, the scores of MSFsc (OR: 0.10, 95% CI: 0.03–0.34, *p* < 0.001), FIRST (OR: 5.03, 95% CI: 1.14–22.24, *p* = 0.03), PSASs (OR: 15.16, 95% CI: 1.93–118.97, *p* = 0.01), PSASc (OR: 4.56, 95% CI: 1.00–20.71, *p* = 0.05), and DBAS (OR: 3.18, 95% CI: 1.06–9.50, *p* = 0.04) were significantly associated. On the other hand, the scores of FIRST (OR: 9.78, 95% CI: 3.01–31.84, *p* < 0.001), PSASs (OR: 2.21, 95% CI: 1.15–4.26, *p* = 0.02), PSASc (OR: 9.60, 95% CI: 3.33–27.63, *p* < 0.001), and DBAS (OR: 1.87, 95% CI: 1.04–3.38, *p* = 0.04) were significantly associated with TRP whereas the MSFsc was not. None of the chronobiological or psychological measures were significantly associated with the presence of the DMS alone subtype.

**Table 2:** Logistic regression analysis of the factors associated with the presence of respective phenotype insomnia.

	OR	95%CI	*p*‐value		OR	95% CI	*p*‐value
DIS				DIS + DMS			
MSFsc	2.01	(1.25 to3.23)	0.00	MSFsc	2.34	(1.33 to 4.12)	0.00
FIRST	2.89	(1.49 to 5.62)	0.00	FIRST	1.66	(0.85 to 3.22)	0.14
PSASs	2.43	(1.34 to 4.40)	0.00	PSASs	1.95	(1.02 to 3.74)	0.04
PSASc	7.23	(3.16 to 16.56)	0.00	PSASc	8.70	(3.27 to 23.14)	0.00
DBAS	2.54	(1.43 to 4.51)	0.00	DBAS	4.56	(2.05 to 10.16)	0.00
DMS				DMS + EMA			
MSFsc	0.79	(0.26 to 2.38)	0.67	MSFsc	0.10	(0.03 to 0.34)	0.00
FIRST	1.33	(0.35 to 5.01)	0.68	FIRST	5.03	(1.14 to 22.24)	0.03
PSASs	0.70	(0.22 to 2.19)	0.54	PSASs	15.16	(1.93 to 118.97)	0.01
PSASc	12254680.89	(0.00 to	0.98	PSASc	4.56	(1.00 to 20.71)	0.05
DBAS	1.38	(0.42 to 4.61)	0.60	DBAS	3.18	(1.06 to 9.50)	0.04
EMA				DIS + EMA			
MSFsc	0.44	(0.24 to 0.81)	0.01	MSFsc	1.77	(0.72 to 4.38)	0.21
FIRST	1.69	(0.84 to 3.40)	0.14	FIRST	2.90	(0.81 to 10.34)	0.10
PSASs	1.67	(0.84 to 3.31)	0.14	PSASs	15.43	(1.95 to 122.37)	0.01
PSASc	4.53	(1.98 to 10.37)	0.00	PSASc	2.46	(0.67 to 8.98)	0.17
DBAS	2.56	(1.27 to 5.15)	0.01	DBAS	2.44	(0.81 to 7.38)	0.11
TRP							
MSFsc	0.82	(0.50 to 1.34)	0.43				
FIRST	9.78	(3.01 to 31.84)	0.00				
PSASs	2.21	(1.15 to 4.26)	0.02				
PSASc	9.60	(3.33 to 27.63)	0.00				
DBAS	1.87	(1.04 to 3.38)	0.04				

*Note*: Age and sex were set as adjustment variables.

Abbreviations: CI, confidence interval; DBAS, Dysfunctional Beliefs and Attitudes about Sleep Scale; DIS, difficulty maintaining sleep; DMS, difficulty initiating sleep; EMA, early morning awakening; FIRST, Ford Insomnia Response to Stress Test; MSFsc, Sleep‐corrected Midsleep on Free Days; OR, odds ratio; PSASc, Pre‐sleep Arousal Scale‐Cognitive; PSASs, Pre‐sleep Arousal Scale‐Somatic; TRP, triplet of DIS, DMS, and EMA symptoms.

### Factors associated with ISI scores in each insomnia subtype

Multiple regression analysis showed that the scores of PSASc and DBAS were significantly associated with ISI scores in the DIS group (*R* ^2^ = 0.17; PSASc, *β* = 0.25, 95% CI: 0.01–0.16, *p* = 0.03; DBAS, *β* = 0.25, 95% CI: 0.00–0.04; *p* = 0.03). However, in the EMA group, only the DBAS score was significantly associated with the ISI score (*R* ^2^ = 0.16, *β* = 0.42, 95% CI: 0.01–0.06, *p* < 0.001). As for DIS + DMS, the DBAS score was significantly associated with the ISI score in DIS + DMS (*R* ^2^ = 0.18, *β* = 0.44, 95% CI: 0.09–0.28, *p* < 0.001) and DIS + EMA (*R* ^2^ = 0.13, *β* = 0.42, 95% CI: 0.00–0.07, *p* =0.05). In the TRP group, both DBAS and PSASs scores were significantly associated with ISI scores (*R* ^2^ = 0.27; DBAS: *β* = 0.42, 95% CI: 0.02–0.07, *p* < 0.001; PSASs: *β* = 0.25, 95% CI: 0.02–0.24, *p* = 0.02) (Table 3). In the group with DMS and that with DMS + EMA, calculation for the analysis could be made due to the shortage of the samples.

**Table 3:** Multiple regression analysis of the factors associated with the scores of the Insomnia Severity Index in respective insomnia subtypes.

	*B*	SE	95% CI	*p* Value
DIS				
PSASc	0.25	0.04	(0.01 to 0.16)	0.03
DBAS	0.25	0.01	(0.00 to 0.04)	0.03
EMA				
DBAS	0.42	0.01	(0.01 to 0.06)	0.00
DIS + DMS				
DBAS	0.44	0.05	(0.09 to 0.28)	0.00
DIS + EMA				
DBAS	0.42	0.02	(0.00 to 0.07)	0.05
TRP				
DBAS	0.42	0.01	(0.02 to 0.07)	0.00
PSASs	0.25	0.06	(0.02 to 0.24)	0.02

*Note*: Age and sex were set as adjustment variables. DMS and DMS + EMA could not be statistically analyzed owing to the small sample size.

Abbreviations: CI, confidence interval; DBAS, Dysfunctional Beliefs and Attitudes about Sleep Scale; DIS, difficulty initiating sleep; DMS, difficulty initiating sleep; EMA, early morning awakening; PSASc, Pre‐sleep Arousal Scale‐Cognitive; PSASs, Pre‐sleep Arousal Scale‐Somatic; SE, standard error; TRP, triplet of DIS, DMS, and EMA symptoms.

### Association of insomnia subtypes with the presence of anxiety and depression

The results of logistic regression analyses after adjusting for demographic variables and the ISI scores are presented in Table 4. Even when the ISI score was set as a covariate, all subtypes were significantly associated with the presence of both anxiety (DIS, OR: 10.27, 95% CI: 6.32–16.69, *p* < 0.001; EMA, OR: 5.75; 95% CI: 3.17–10.45, *p* < 0.001; DIS + DMS, OR: 11.22, 95% CI: 6.29–20.33, *p* < 0.001; DIS + EMA, OR: 6.04, 95% CI: 2.55–14.31, *p* < 0.001; DMS + EMA, OR: 9.56, 95% CI: 3.78–24.18, *p* < 0.001; TRP, OR: 12.92, 95% CI: 7.15–23.34, *p* < 0.001) and depression (DIS, OR: 3.89, 95% CI: 2.26–6.72, *p* < 0.001; EMA, OR: 5.11, 95% CI: 2.16–12.09, *p* < 0.001; DIS + DMS, OR: 4.22, 95% CI: 2.20–8.11, *p* < 0.001; DIS + EMA, OR: 8.41, 95% CI: 1.96–36.06, *p* < 0.001; DMS + EMA, OR: 2.25, 95% CI: 0.88–5.71, *p* < 0.001; TRP, OR: 7.41, 95% CI: 3.37–16.26, *p* < 0.001).

**Table 4:** Logistic regression analysis of the factors associated with the presence of anxiety/depression.

	OR	95% CI	*p* Value
Anxiety			
DIS	10.27	(6.32 to 16.69)	0.00
DMS	5.36	(1.75 to 16.45)	0.00
EMA	5.75	(3.17 to 10.45)	0.00
DIS + DMS	11.22	(6.29 to 20.03)	0.00
DIS + EMA	6.04	(2.55 to 14.31)	0.00
DMS + EMA	9.56	(3.78 to 24.18)	0.00
TRP	12.92	(7.15 to 23.34)	0.00
Depression			
DIS	3.89	(2.26 to 6.72)	0.00
DMS	1.96	(0.60 to 6.42)	0.27
EMA	5.11	(2.16 to 12.09)	0.00
DIS + DMS	4.22	(2.20 to 8.11)	0.00
DIS + EMA	8.41	(1.96 to 36.06)	0.00
DMS + EMA	2.25	(0.88 to 5.71)	0.09
TRP	7.41	(3.37 to 16.26)	0.00

*Note*: Age, sex, and Insomnia Severity Index score were set as adjustment variables. DMS was unable to perform statistical analysis due to the small sample size.

Abbreviations: CI, confidence interval; DIS, difficulty initiating sleep; DMS, difficulty maintaining sleep; EMA, early morning awaking; OR, odds ratio; TRP, triplet of DIS, DMS, and EMA symptoms.

## DISCUSSION

Previous reports have shown that the presence of several insomnia subtypes is influenced by certain chronotypes, that higher FIRST score is associated with the vulnerability of the occurrence of insomnia symptoms,^10^ and that a higher DBAS score is associated with the formation of insomnia chronicity.^15^, ^30^ In the present study, significant differences were observed in the factors associated with the presence and severity of insomnia disorder in respective subtypes. Particularly, the association of chronotype scores differed among the subtypes. Namely, DIS and DIS + DMS were significantly associated with a tendency toward eveningness, whereas EMA and EMA + DMS were associated with morningness. These results obtained in our epidemiological study are consistent with the results of experimental studies conducted by Lack et al.^7^, ^8^ In contrast to a previous study,^9^ the TRP group was neither morningness‐ nor eveningness‐oriented in the present study. One possible reason for this discrepancy is that Bjorøy et al.^9^ used a self‐reported questionnaire for circadian preference, while this study employed the MCTQ, which is a validated chronotype measure. In any case, the presence of TRP was thought to be strongly impacted by psychological factors as manifested in various measures. DIS + DMS showed the same chronotype pattern as DIS, and EMA + DMS showed the same chronotype pattern as EMA. This suggests that when DIS and DMS merge, they show characteristics of DIS, and when EMA and DMS merge, they reflect characteristics of EMA. In contrast, when DIS and EMA merge, the effects of the two chronotypes are expected to cancel each other out.

In the present study, the FIRST score was significantly associated with the presence of chronic insomnia but not with the severity of the disorder, as the chronotype measures did. This result is in line with previous studies showing that the FIRST score is a vulnerable factor in insomnia.^10^ Thus, the FIRST score may contribute to the development of insomnia but does not play a role as a maintenance factor. If this is the case, FIRST should be used as a marker for predicting the onset or relapse of chronic insomnia.

In this study, the PSASc score was strongly associated with the presence of mostly all insomnia symptom subtypes. This finding was in line with the results of the study by Kalmbach et al.^31^ demonstrating that heightened cognitive arousal at night had significant impacts on the prolongation of either sleep latency, latency to persistent sleep, and wake after sleep onset, as well as a decrease in sleep efficiency and total sleep time in people with chronic insomnia. Moreover, the present study revealed that the PSASc score was associated with insomnia severity in the DIS groups. This result did not contradict the idea that the decrease in PSASc could be a mediator of improvement in insomnia severity, as reported by Parsons et al.^16^ Interestingly, PSASs scores appeared to be specifically associated with insomnia severity in TRP. The reason for this finding remains unclear. However, considering that this group had the highest HADSa, increased anxiety might be related with somatic arousal.^32^

In this study, DBAS scores seemed to be associated with increased insomnia severity in mostly all subtypes, impressing that dysfunctional beliefs and attitudes are related to the development of chronic insomnia.^16^ Moreover, the association of DBAS scores even with the presence of all chronic insomnia subtypes possibly suggests that it is also involved in the pathological mechanism of relatively early stages of the disorder. However, this finding could be attributed to the fact that the present study included chronic insomnia with symptoms lasting longer than 3 months.

Of note, all insomnia subtypes were associated with increased HADSa and HADSd scores, and this association was not different among the subtypes except for DMS. These results are inconsistent with those of previous studies. For instance, Taylor et al.^6^ showed that individuals with a combination of DIS and DMS had higher depression scores than those with symptoms of only DIS or DMS and that there was no significant subtype‐specific difference in the anxiety score. The reason for this phenomenon is unclear; however, our findings highlight that single‐symptom subtypes, even EMA, have a certain pathological importance. Of note, insomnia subtypes were positively associated with both HADSa and HADSd scores, even after controlling for the ISI score, suggesting that the presence of almost all subtypes may contribute to the development of depression or anxiety, independent of the severity of the disorder.

This study had several limitations. First, although this study classified participants with chronic insomnia based on the ICSD‐3 diagnostic criteria, the classification was not made with face‐to‐face interviews, possibly leading to false results. Second, this study was a web‐based survey that might have caused sampling biases such that participants were likely to have more sleep problems or shorter sleep durations in general.^33^ Third, although participants' self‐reported midpoint of sleep was employed for estimating their circadian phase in this study, actigraphy recording and measurement of nighttime melatonin secretion would be necessary to accurately evaluate the circadian rhythm characteristics. Fourth, some analyses could not be sufficiently made particularly on the DMS and DMS + EMA subtypes because of the small sample size. Although the proportion of individuals with insomnia disorder in this study did not deviate from previous epidemiological studies,^2^ a larger sample size would be needed for classification by symptomatic subtypes. Finally, because this study was conducted as a cross‐sectional survey, causal relationships between insomnia subtypes and descriptive measures remain inconclusive. Therefore, a longitudinal follow‐up study is required to confirm the results of this study.

In conclusion, the presence of chronic insomnia may be influenced by both psychological factors and chronotypes, while the severity of insomnia disorder may be influenced only by psychological factors. Additionally, all insomnia subtypes may contribute to the development of depression and anxiety. Therefore, to improve the treatment outcome of chronic insomnia, appropriate techniques should be selected for each of the factors identified in this study as contributing to the presence and aggravation of the disorder.

## AUTHOR CONTRIBUTIONS

Isa Okajima made substantial contributions to the conception of the work. Yuichi Inoue made significant contributions to the data analysis and interpretation. Masumi Osao made significant contributions to the design of the work and interpretation of the data. Isa Okajima and Yuichi Inoue drafted the original manuscript. All authors substantially contributed to the revision of the manuscript drafts. All authors have approved the submitted version of the manuscript and have agreed to be accountable for any part of the work.

## CONFLICT OF INTEREST STATEMENT

Y. I. received honoraria for speaking from Eisai Co. Ltd. and MSD K.K. I. O. received grants from NEC Solution Innovators Co., Ltd. and Infocom Co.; lecture fees from Otsuka Pharmaceutical Co., Ltd., MSD LLC, and Eisai Co., Ltd.; and consultation fees from NEC Solution Innovators Co., Ltd. and Suntory Wellness Ltd. for projects unrelated to the submitted work. M. O. declares no conflicts of interest. The funders had no role in the design of the study; in the collection, analyses, or interpretation of data; in the writing of the manuscript; or in the decision to publish the results.

## ETHICS APPROVAL STATEMENT

The study protocol was approved by the Ethics Committee of Tokyo Medical University, Tokyo, Japan (registration no. 3307).

## PATIENT CONSENT STATEMENT

All study participants provided informed consent prior to participation, in accordance with the Declaration of Helsinki.

## CLINICAL TRIAL REGISTRATION

N/A

## Data Availability

The datasets generated and analyzed during the current study are available from the corresponding author on reasonable request.
